# Recombinant Activated Factor VII (rFVIIa) in the Management of Major Obstetric Haemorrhage: A Case Series and a Proposed Guideline for Use

**DOI:** 10.1155/2009/364843

**Published:** 2010-02-03

**Authors:** Charlotte Bomken, Sue Mathai, Tina Biss, Andrew Loughney, John Hanley

**Affiliations:** ^1^Department of Haematology, The Newcastle upon Tyne Hospitals NHS Trust, Freeman Hospital, Freeman Road, High Heaton, Newcastle-upon-Tyne NE7 7DN, UK; ^2^Department of Obstetrics, The Newcastle upon Tyne Hospitals NHS Trust, Newcastle-upon-Tyne NE1 4LP, UK

## Abstract

Major obstetric haemorrhage remains a significant cause of maternal morbidity and mortality. Previous case reports suggest the potential benefit of recombinant activated factor VII (rFVIIa: NovoSeven^R^) as a haemostatic agent. We performed a retrospective review of the use of rVIIa in major obstetric haemorrhage in the Northern Region between July 2004 and February 2007. Fifteen women received rFVIIa. The median patient age was 34 years. Major haemorrhage occurred antepartum (5 patients), intrapartum (1), and postpartum (9). All women received an initial dose of 90 mcg/kg rFVIIa and one received 2 further doses. Bleeding stopped or decreased in 12 patients (80%). Additional measures included antifibrinolytic and uterotonic agents, Rusch balloon insertion, uterine curettage/packing, and vessel embolisation. Eight patients required hysterectomy. All women survived to discharge from hospital. No adverse events, including thrombosis, were recorded. This study provides further support for the safety and efficacy of rFVIIa as adjunct therapy in major obstetric haemorrhage.

## 1. Introduction

Obstetric haemorrhage remains a significant cause of maternal morbidity and mortality. Worldwide, life-threatening haemorrhage occurs in 10% of all live births [1]. In the UK, obstetric haemorrhage has a mortality rate of 8.5 per million maternities; the most recent triennial report of the Confidential Enquiry into Maternal and Child Health (2000–2002) identified 17 maternal deaths due to obstetric haemorrhage. Care was described as potentially substandard in 71% of these cases. This mostly referred to organisational problems, in particular deficiencies in interprofessional communication [1]. The management of major obstetric haemorrhage remains challenging, requiring both surgical and medical interventions, sometimes resulting in hysterectomy. A multidisciplinary team approach involving clear communication between pathology laboratory staff, obstetricians, midwives, haematologists, and radiologists is therefore essential, and clear written guidelines for intervention can be helpful.

In recent years, the off-licence use of rFVIIa as a haemostatic agent in the management of life-threatening haemorrhage in a variety of clinical settings has become well recognised [2–8]. There are several published case reports and small case series documenting the effective use of rFVIIa in the management of major obstetric haemorrhage [9–15]. However, large case series and randomised controlled trials have not been conducted. We present our experience of the administration of rFVIIa in a consecutive cohort of women with major obstetric haemorrhage in the Northern region of England and propose a guideline for appropriate use in this setting.

## 2. Methods

### 2.1. Protocol for the Administration of rFVIIa

Administration of rFVIIa for major obstetric haemorrhage was according to a regional consensus protocol. RFVIIa was indicated if significant bleeding (at an estimated rate of greater than 200 mL/hr) continued despite optimal medical/surgical measures and blood component replacement therapy, to achieve a platelet count greater than 50 × 10^9^/L, a fibrinogen >1.0 g/L, and a prothrombin time and activated partial thromboplastin time <1.5 times greater than the upper limit of normal. Authorisation by a Consultant Haematologist was required prior to use of rFVIIa. A dose of 90 mcg/kg was recommended, with a second dose 3 hours later if an initial response was not obtained. We proposed that a maximum of two doses should be given, with further doses being given only in exceptional circumstances, for example, where the obstetrician, haematologist, and anaesthetist agreed that further doses might be useful, particularly if exsanguination seemed likely. Platelet count, prothrombin time (PT), activated partial thromboplastin time (APTT), and Clauss fibrinogen were measured immediately before, and 15 minutes after, administration of the first dose. Bilateral lower limb Doppler ultrasound was performed after 3–5 days to assess the presence of deep venous thrombosis (DVT).

### 2.2. Data Collection

Data were obtained by retrospective review of patients' case notes and laboratory records. Details of patient age, gestation, circumstances leading to the haemorrhagic event, dose of rFVIIa and number of doses administered, and additional measures taken to achieve haemostasis were recorded. Clinical response (bleeding unchanged, bleeding decreased, or bleeding stopped) was assessed subjectively by the attending obstetrician or anaesthetist 1 hour after the first dose of rFVIIa. Laboratory results (platelet count, PT, APTT, and Clauss fibrinogen), and details of blood components transfused during the 24 hours before and after rFVIIa administration were noted. Case notes were examined for any reported thrombotic events occurring within 7 days of administration of rFVIIa.

## 3. Statistical Analysis

Coagulation parameters and blood component use pre and post rFVIIa administration were analysed using Wilcoxon signed rank test for paired nonparametric data. A *P* value of <.05 was considered to be significant.

## 4. Results

### 4.1. Patient Demographics

Fifteen women received rFVIIa for major obstetric haemorrhage over a 31-month period between July 2004 and February 2007 (Table 1). Median age was 34 years (range: 20–46 years). Fourteen women had singleton pregnancies, one had a twin pregnancy. One patient (Patient 9) was a Jehovah's Witness who did not consent to the administration of blood products. 

### 4.2. Indication for rFVIIa

Indications for the use of rFVIIa were as follows: antepartum haemorrhage, 5 patients; intrapartum haemorrhage, 1 patient; and postpartum haemorrhage, 9 patients. Cause of bleeding was identified as follows: uterine atony, 6 patients; complications following Caesarean section, 2 patients; placenta praevia, 3 patients; uterine perforation, 1 patient; abruption, 2 patients; and uterine eversion, 1 patient.

### 4.3. Dosing and Timing of Administration of rFVIIa

All patients received the recommended dose of rFVIIa of 90 mcg/kg. The median time from start of bleeding to administration of the first dose of rFVIIa was 4 hours and 5 minutes (range 1 hour–20 hours 33 minutes). 14 patients received 1 dose of rFVIIa and 1 patient (patient 7) received 3 doses. 

### 4.4. Response to rFVIIa: Clinical Response, Coagulation Parameters, and Blood Transfusion Requirements

**Figure 1:**
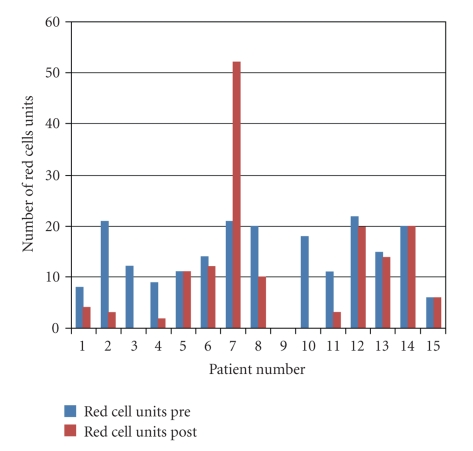
Red cell transfusion requirements within 24 hours before and after the administration of rVIIa for major obstetric haemorrhage.

A clinical response to rFVIIa (defined as bleeding decreased or stopped at 1 hour post administration) was seen in 12/15 (80%) patients. The median number of units of blood components transfused following rFVIIa was observed to be lower than before rFVIIa (Table 2). However, a significant difference was only demonstrated for transfusion of red cells (*P* = .034) (Figure 1). The relatively low number of nonresponding patients did not allow for subgroup analysis of responders versus nonresponders.

Coagulation data were collected for all patients. Median pre and posttreatment PTs were 15 seconds (range: 13–29) and 10 seconds (range: 6.8–28); *P* = .007. Median pre- and posttreatment APTTs were 50 seconds (range: 21–160) and 33 seconds (range: 26.5–150); *P* = .025. No significant change was seen in platelet count (*P* = .188) or Clauss fibrinogen (*P* = .073) (Table 3).

### 4.5. Additional Measures Taken to Achieve Haemostasis

Prior to rFVIIa administration, additional measures to control blood loss included blood product support, uterotonic agents, antifibrinolytic agents, uterine packing, insertion of Rusch balloon, B-lynch suture, and vessel embolisation/ligation. A hysterectomy was performed in 4 patients prior to (1 patient) or concurrent with (3 patients) the administration of rFVIIa. Following the administration of rFVIIa, additional measures were required to achieve haemostasis in 7 patients. In 4 of these patients, these measures included a total (3 patients) or subtotal (1 patient) hysterectomy.

## 5. Outcome

No clinical evidence of venous or arterial thrombosis was identified in any of the fifteen women who received rFVIIa. Of the nine women with a documented lower limb Doppler ultrasound result, no radiological evidence of deep vein thrombosis was recorded. All patients survived to discharge from hospital.

## 6. Discussion

Although the death rate due to obstetric haemorrhage has fallen over the last 40 years, the most recent triennial report for the United Kingdom has seen an increase in maternal mortality due to haemorrhage from 3.3 to 8.5 per million maternities [1]. Obstetric haemorrhage can progress rapidly and is strongly associated with the development of disseminated intravascular coagulation (DIC) [16]. Delayed correction of DIC is associated with a significant increase in morbidity and mortality. Rapid correction of coagulopathy with ongoing regular monitoring of coagulation status is often difficult to coordinate in the setting of acute life-threatening obstetric haemorrhage. However, this is vital to the successful management of such cases. It has therefore been proposed that the management of major obstetric haemorrhage should take place in conjunction with a haematologist to ensure the early identification and rapid correction of DIC, with the subsequent administration of rFVIIa where appropriate, and the frequent monitoring required in bleeding patients [16].

rFVIIa works locally by directly forming a complex with exposed tissue factor on subendothelial cells. This complex activates factors IX and X leading to the generation of small amounts of thrombin. This in turn activates factors V and VIII and platelets perpetuating thrombin generation and resulting in a “thrombin burst.” The action of rFVIIa is limited to the site of tissue injury and tissue factor exposure. This is particularly useful in the obstetric setting where there is often bleeding from a large raw area of exposed tissue. Action of rFVIIa is dependant on the presence of adequate numbers of circulating platelets and adequate fibrinogen concentration. Previous reports have shown that the improvements in laboratory markers of DIC, by the administration of blood components prior to the administration of rFVIIa, are associated with a better response to rFVIIa treatment [4]. It is also recognised that correction of acidosis is an important aspect in the success of rFVIIa. Measurement of pH is not a routine investigation in the management of obstetric haemorrhage within our region. We have therefore not included pH data in this study.

This case series documents a clinical response (bleeding stopped or decreased) to rFVIIa in 80% of patients, which is comparable to previous documented clinical response rates described in case reports and small series in obstetric haemorrhage [9–15]. It is also comparable to the response to rFVIIa reported in life-threatening haemorrhage from all causes, where the median response rate is reported as 74% [2–8]. There have been no large case series or randomised controlled trials on the use of rFVIIa in obstetric haemorrhage. However, a recently published case series of 26 patients reviewing the use of rFVIIa in post-partum haemorrhage, documented a good response (bleeding <1000 mL post administration) or moderate response (ongoing blood loss >1000 mL without the need for additional surgical or radiological intervention) to rFVIIa treatment in 61% of patients [9]. We believe that even a reduction in blood loss that is sufficient to allow additional procedures to achieve complete haemostasis is clinically significant.

The use of blood, FFP, platelets, and cryoprecipitate was reduced in this series in the majority of patients following the administration of rFVIIa, but not in all patients. Where large volume blood loss occurred, adequate red cell replacement may take time to achieve, with patients requiring ongoing transfusion despite an observed decrease in bleeding. The number of red cell units required may therefore be a poor indicator of response to rFVIIa. The relatively small number of patients makes this series vulnerable to the effects of single patients. 1 patient in our series (patient 7) received rFVIIa for massive intrapartum haemorrhage following intrauterine foetal death at 30 weeks gestation with the development of subsequent DIC, requiring emergency caesarean section. Despite blood product support, uterotonic agents and antifibrinolytic agents, the coagulopathy worsened and she developed increasing blood product requirements. There was a documented reduction in blood loss initially following administration of rFVIIa. However, bleeding subsequently worsened and the patient required a hysterectomy. The patient received in addition a further 2 doses of rFVIIa, with an observed decrease in bleeding after each dose, and required arterial embolisation of 4 vessels with subsequent haemostasis 12 hours after her original presentation. Despite the effects of this important case, our data suggest an overall decrease in blood component use following the administration of rFVIIa. To those patients who responded well to rFVIIa, this is clinically very important. It reduces their exposure to blood products and thereby reduces the risk of transfusion reaction, transfusion transmitted infection, and alloantibody formation, and prevents worsening of coagulopathy by the effects of dilution, hypothermia, and acidosis.

In addition to blood component support, all patients in this series required further obstetric or surgical measures to control bleeding. In 8 patients, vessel ligation, embolisation, or hysterectomy was performed prior to the administration of rFVIIa. Bleeding rate was unchanged in 2 of these patients following rFVIIa administration and both patients required a hysterectomy. However, these 2 patients had additional risk factors (coagulopathy, administration of >10 units of blood, acidosis and hypothermia) prior to rFVIIa administration. All of these risk factors have been shown to be associated with a worse outcome following the administration of rFVIIa [2–8]. A further 2 patients required vessel embolisation following rFVIIa administration. Both patients suffered uterine atony (1 following uterine eversion) which failed to settle with uterotonic agents. In both patients, hysterectomy was performed prior to the administration of rFVIIa but bleeding continued. In both patients a response to rFVIIa was documented initially but bleeding subsequently worsened. Both patients were found to have bleeding from major blood vessels. One patient required radiological embolisation to a bleeding pelvic artery and subsequent re-laparotomy before bleeding was controlled. The second patient received 3 doses of rFVIIa prior to embolisation of both iliac arteries, right circumflex artery and right internal pudendal artery. 

Emergency hysterectomy is a procedure associated with a risk of significant long-term maternal morbidity [17, 18]. Avoidance, where possible, significantly reduces morbidity and preserves future fertility. As an adjunct to obstetric and surgical measures, we believe that consideration of rFVIIa use should be given early, prior to hysterectomy or vessel ligation. Proposed guidance for the use of rFVIIa in major obstetric haemorrhage is detailed in Table 4. It is clear that rFVIIa is unlikely to be effective as a sole measure in the presence of bleeding from large vessels. It may however slow the rate bleeding and prevent further deterioration by delaying the onset of DIC and by reducing the number of units of blood transfused, thus reducing hypothermia, acidosis, and dilutional coagulopathy.

No thrombotic events were documented in our series despite the relative prothrombotic state of pregnancy. Many of the thrombotic events seen in association with rFVIIa are arterial and occur in patients with preexisting vascular risk factors [19], and reports have documented a low thromboembolic risk in previously healthy patients with major haemorrhage even in the presence of DIC [8, 20]. Nevertheless, it was very reassuring that this potentially significant event was not observed in our series, providing further evidence for the safety of rFVIIa in major obstetric haemorrhage.

## 7. Conclusions

This report provides evidence to support the ongoing use of rFVIIa in the management of life-threatening obstetric haemorrhage as a successful adjunct to optimal medical and obstetric intervention. It is unlikely that we will see randomised control trial data comparing the use of rFVIIa with placebo given the practical difficulties of formally consenting and then randomising patients who are experiencing life-threatening obstetric haemorrhage. It would be difficult to justify withholding a treatment that can be efficacious in obstetric haemorrhage in favour of placebo agents in this setting. We therefore rely on data collected from case series. Larger series are required in order to clarify the benefit of rFVIIa use and to allow the creation of clear guidelines for the established but off-licence use of this agent. To collate and analyse data on the use of rVIIa in obstetric haemorrhage, the Northern Europe Factor VIIa in Obstetric Haemorrhage (NEFOH) Registry has been formed. This allow for the collection of information on a larger number of cases with the intention of providing further guidance on the future use of rVIIa in the management of obstetric haemorrhage.

## Figures and Tables

**Table 1:** Clinical indication and response to rFVIIa following major obstetric haemorrhage. rFVIIa—recombinant activated factor VII, TAH—total abdominal hysterectomy.

Pt no.	Maternal age (years)	Cause of bleeding	Time from start of bleeding to rFVIIa administration (hours:minutes)	No. of rFVIIa doses administered	Measures taken to secure haemostasis before rFVIIa	Measures taken to secure haemostasis after rFVIIa	Blood loss following rFVIIa
1	33	Uterine atony	12:35	1	Uterotonics, curettage, uterine packing	Nil	Stopped
							
2	34	Post emergency Caesarean section	20:33	1	Aprotinin, ligation of broad ligament bleed point	Bilateral iliac artery ligation, splenectomy	Stopped
							
3	30	Retained placenta, uterine perforation	2:00	1	TAH (rFVIIa given intraoperatively)	Nil	Stopped
							
4	30	Placental abruption	9:15	1	Uterotonics	Nil	Stopped
							
5	38	Uterine atony following exit procedure	1:59	1	Uterotonics	Nil	Decreased
							
6	20	Uterine atony, vaginal lacerations	2:57	1	Uterotonics, Rusch balloon	Nil	Decreased
7	34	Intrapartum bleeding with uterine atony following emergency Caesarean section	5:10	3	Uterotonics, Rusch balloon, TAH (1st dose rFVIIa given intraoperatively)	2nd rVIIa followed by 4 vessel embolisation, 3rd rFVIIA followed by repair bladder tear and further embolisation	Decreased
							
8	46	Placenta accreta and placenta praevia,	4:35	1	TAH	Laparotomy but no bleeding point found.	Decreased
							
9	32	Placenta praevia (Jehovah's Witness)	8:00	1	Uterotonics, antifibrinolytics, B-Lynch suture	TAH	Decreased
							
10	36	Placental abruption during road traffic accident	5:00	1	Uterotonics, laparotomy and intra-abdominal packing	Nil	Decreased
							
11	38	Placenta praevia	3:05	1	Balloon tamponade, vaginal packing	Nil	Decreased
12	36	Uterine eversion	1:00	1	Uterotonics, uterine packing, vessel embolisation, TAH (rFVIIa given intraoperatively)	Repeat laparotomy and abdominal packing	Decreased
							
13	21	Placenta praevia	3:30	1	Uterotonics, antifibrinolytics	TAH	Unchanged
							
14	43	Post elective Caesarean section, broad ligament tear	4:05	1	Laparotomy and repair of tear	Subtotal hysterectomy	Unchanged
							
15	20	Uterine atony	3:00	1	Uterotonics, uterine packing, B-Lynch suture, iliac artery ligation,	TAH	Unchanged

**Table 2:** Blood product requirements before and after the administration of rFVIIa for major obstetric haemorrhage.

Pt no.	FFP transfused		Cryoprecipitate		Platelets transfused		Red cells transfused	
	(units)		transfused (units)		(pools)		(units)	
	Pre	Post	Pre	Post	Pre	Post	Pre	Post
1	2	0	10	0	0	1	8	4
2	12	0	10	0	2	0	21	3
3	4	0	9	0	2	0	12	0
4	4	0	5	5	2	0	9	2
5	4	4	0	10	0	3	11	11
6	4	0	10	0	0	2	14	12
7	7	31	8	42	1	12	21	52
8	10	6	3	0	2	2	20	10
9	0	0	0	0	0	0	0	0
10	12	0	20	10	2	0	18	0
11	4	0	0	0	0	0	11	3
12	6	15	10	10	2	5	22	20
13	8	7	0	10	2	2	15	14
14	10	0	10	0	1	0	20	10
15	4	5	30	0	1	0	6	6

Median	4	0	9	0	1	0	14	6

*P*-value*	0.127		0.413		0.700		0.034	

*Wilcoxon signed-rank test is shown.

**Table 3:** Coagulation parameters before and after the administration of rFVIIa for major obstetric haemorrhage. Normal ranges: Prothrombin time 10–13 seconds; Activated partial thromboplastin time 25–37 seconds; Fibrinogen 2.1–4.8 seconds.

Pt no.	Prothrombin time		Activated partial		Platelet count		Fibrinogen	
	(seconds)		thromboplastin time (seconds)		(×10^9^/L)		(g/L)	
	Pre	Post	Pre	Post	Pre	Post	Pre	Post
1	14	9	30	30	93	96	1.7	1.5
2	21	13	49	33	106	85	1.4	2.6
3	16	10	52	29	129	103	0.9	2.4
4	15	10	34	25	89	93	0.9	1.9
5	16	11	64	57	160	125	1.3	1.7
6	18	11	60	31	68	48	0.4	1.4
7	29	12	160	93	6	22	0.6	0.9
8	15	10	34	37	48	45	1.5	2.1
9	15	9	39	30	89	119	2	2.1
10	15	9	60	30	64	69	0.9	3.6
11	15	9	50	37	76	62	0.9	0.8
12	13	10	50	35	35	57	2.1	2.3
13	14	28	31	150	308	28	4.1	1.3
14	9.2	6.8	101	41	51	34	1.9	3.36
15	16.4	10.6	21	26.5	327	88	2.6	2

Median	15	10	50	33	89	69	1.4	2.0

*P*-values*	0.007		0.025		0.188		0.073	

*Wilcoxon signed-rank test is shown.

**Table 4:** 

**Guidance for the use of rFVIIa in major obstetric haemorrhage**
(i) Use of rFVIIa should be considered in major obstetric haemorrhage:
-which continues despite optimal blood product replacement and obstetric measures.
-where uterine artery ligation/embolisation or hysterectomy are under consideration.
-with clinical haemostatic failure (i.e., oozing from multiple sites), where there is unavoidable delay in the provision of
blood products.
-in women who refuse blood or blood components, for example, Jehovah's Witness
(ii) A dose of 90 *μ*g/kg is recommended
(iii) Use of rFVIIa should be authorised by a Consultant Haematologist or Consultant Obstetric Anaesthetist prior to administration.
(iv) A single standard dose should be kept in delivery suite to facilitate rapid administration in appropriate circumstances.
(v) Use of rFVIIa should not be seen as an alternative to surgical haemostasis or correction of coagulopathy with blood products. Before
administration of rFVIIa, the following laboratory indices are desirable;
–Prothrombin time < 1.5 × upper limit of normal
–Clauss fibrinogen > 1.0 g/L
–Platelet count > 50 × 10^9^/L
(vi) Along with the above laboratory indices a pH > 7.1 is also desirable for optimal effect.
(vii) Further doses should only be given in exceptional circumstances where agreed by the multidisciplinary team, for example, where
exsanguination seems likely.
